# *QuickStats: *Percentage Distribution* of Cigarette Smoking Status^†^ Among Current Adult E-Cigarette Users,^§^ by Age Group — National Health Interview Survey, United States, 2021^¶^

**DOI:** 10.15585/mmwr.mm7210a7

**Published:** 2023-03-10

**Figure:**
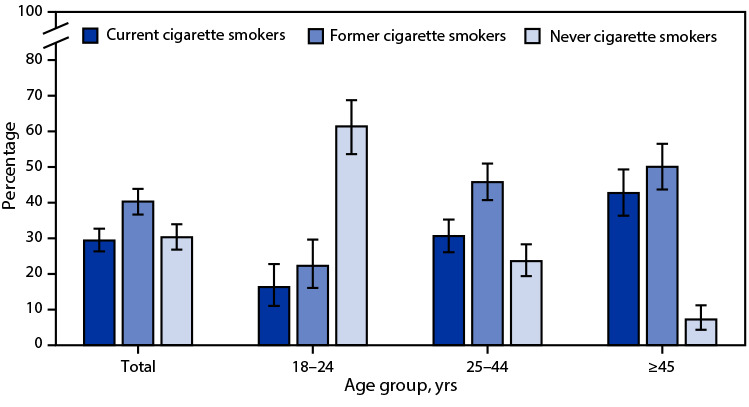
In 2021, 4.5% of U.S. adults were current e-cigarette users. Among adult e-cigarette users overall, 29.4% also were current cigarette smokers, 40.3% were former cigarette smokers, and 30.3% had never been cigarette smokers. Among e-cigarette users aged 18–24 years, 16.3% were current smokers, 22.3% were former smokers, and 61.4% had never been cigarette smokers. Among those aged 25–44 years, 30.6% were current smokers, 45.8% were former smokers, and 23.6% had never smoked cigarettes. Among those aged ≥45 years, 42.7% were current smokers, 50.1% were former smokers, and 7.2% had never smoked cigarettes. Younger e-cigarette users were more likely to have never smoked cigarettes, and older e-cigarette users were more likely to be current or former cigarette smokers.

**Source:** National Center for Health Statistics, National Health Interview Survey, 2021. https://www.cdc.gov/nchs/nhis/index.htm

For more information on this topic, CDC recommends the following link: https://www.cdc.gov/tobacco/basic_information/e-cigarettes/index.htm

